# The probable role and therapeutic potential of the PI3K/AKT signaling pathway in SARS-CoV-2 induced coagulopathy

**DOI:** 10.1186/s11658-022-00308-w

**Published:** 2022-01-11

**Authors:** Mohammad Rafi Khezri, Reza Varzandeh, Morteza Ghasemnejad-Berenji

**Affiliations:** 1 Department of Pharmacology and Toxicology, Faculty of Pharmacy, Urmia University of Medical Sciences, Sero Road, 5715799313 Urmia, Iran; 2 Research Center for Experimental and Applied Pharmaceutical Sciences, Urmia University of Medical Sciences, Urmia, Iran

**Keywords:** SARS-CoV-2, Coagulation, COVID-19, PI3K/AKT

## Abstract

Coronavirus disease 2019 (COVID-19), which is caused by severe acute respiratory syndrome coronavirus-2 (SARS-CoV-2), is associated with a high mortality rate. The majority of deaths in this disease are caused by ARDS (acute respiratory distress syndrome) followed by cytokine storm and coagulation complications. Although alterations in the level of the number of coagulation factors have been detected in samples from COVID-19 patients, the direct molecular mechanism which has been involved in this pathologic process has not been explored yet. The PI3K/AKT signaling pathway is an intracellular pathway which plays a central role in cell survival. Also, in recent years the association between this pathway and coagulopathies has been well clarified. Therefore, based on the evidence on over-activity of the PI3K/AKT signaling pathway in SARS-CoV-2 infection, in the current review, the probable role of this cellular pathway as a therapeutic target for the prevention of coagulation complications in patients with COVID-19 is discussed.

## Background

Coronavirus disease 2019 (COVID-19) is characterized by acute respiratory distress syndrome due to hyperactivity of the immune system [1]. In addition, there are several other complications induced by severe acute respiratory syndrome coronavirus-2 (SARS-CoV-2), such as neurological symptoms [2] and coagulopathies, which have received a lot of attention [3, 4]. Regarding the association between SARS-CoV-2 infection and coagulopathies, different studies have examined the level of factors involved in coagulopathy in COVID-19 patients. In this case, elevated levels of tissue factor (TF), thrombin [5], increased platelet activation [6], and decreased fibrinogen levels [7] have been detected in patients with COVID-19. In addition to the mentioned factors, the association between other elevated factors in COVID-19 patients, such as inflammatory cytokines, angiotensin II (Ang II), and blood clotting, has been considered (reviewed in [8]).

The phosphatidyl-inositol-3-kinase (PI3K)/AKT signaling pathway is a major regulator of cell biology. It regulates different aspects of cell survival such as protein synthesis, apoptosis inhibition, and cell proliferation [9, 10]. The PI3K/AKT signaling pathway has been studied extensively for its role in cancer progression. Furthermore, in recent years the link between this cellular pathway and blood clot generation has been reported in several studies [11, 12]. Recently in the study by Pelzl et al. [13] the activation status of platelets and PI3/AKT signaling in COVID-19 patients were analyzed. In this in vitro study the functionality was evaluated by platelet adhesion ability on fibrinogen with PI3K and AKT inhibitors. Interestingly, the results of this study showed that the inhibition of AKT as well as PI3K could prevent the enhanced activation and adhesion of platelets on fibrinogen as well as pro-coagulant platelet formation in sera of COVID-19 patients. According to this study, inhibiting PI3K/AKT phosphorylation could be a promising strategy to prevent onset of thrombosis in patients with severe COVID-19. In this regard, here we review the role of the PI3K/AKT pathway in regulating blood coagulation for its potential to be a suitable therapeutic target in coagulopathy inhibition in COVID-19 patients.

### The probable mechanism of PI3K/AKT pathway over-activation in SARS-CoV-2 infection

It has been shown that the PI3K/AKT/mTOR pathway plays a crucial role in the pathogenesis of MERS-CoV, and inhibition of this pathway prevents MERS-CoV proliferation in vitro [14]*.* Although alterations in the activity of the PI3K/AKT signaling pathway in biopsies from COVID-19 patients have not been studied so far, studies on the effect of the virus on this pathway have been performed. For instance, it has been reported that the PI3K/AKT signaling pathway could be phosphorylated by the N protein of SARS-CoV, causing the establishment of persistent SARS-CoV infection in Vero E6 cells [15, 16]. Furthermore, it has been elucidated that capivasertib, a PI3K/AKT signal pathway inhibitor, restricts SARS-CoV-2 entry to Vero cells [17]. Also, it has been demonstrated that infection of a human hepatocyte-derived cellular carcinoma cell line, Huh7, by SARS-CoV-2 leads to over-activation of the PI3K/AKT pathway [18]. In this study it was shown that AKT inhibition by MK-2206 suppresses new virus production in these cells. Dactolisib, another PI3K inhibitor, has been demonstrated to affect SARS-CoV-2 production in cardiomyocytes [19]. In this study, it was found that dactolisib, AZD2014, and torin2, which target the PI3K/AKT1/mTOR pathway, can be investigated as therapeutic options against COVID-19. In addition to the mentioned studies, the association between the PI3K/AKT pathway and SARS-CoV-2 receptors, furin and CD147, in platelets should be discussed. The expression of both receptors has been shown on platelets in different studies [20, 21]. Interestingly, it has been elucidated that extracellular cyclophilin A induces the PI3K/AKT pathway activation by mediating CD147, leading to activation of platelets, promotion of their adhesion and thrombus formation [22]. This study can be generalized to SARS-CoV-2 induced infection, so it is probable that the effect of this virus on platelet CD147 may have the same results, but further studies are required to prove this claim. Regarding the other SARS-CoV-2 receptor, furin, it has been demonstrated that furin is closely associated with PI3K/AKT signaling activation [23]. In addition to over-activation of the PI3K/AKT signaling pathway by SARS-CoV-2, the role of this pathway in SARS-CoV-2 entry to the host cells can be examined. In this case, it has been elucidated that endocytosis of the SARS-CoV-2 occurs via a clathrin-mediated pathway [24]. On the other hand, it is clearly understood that the PI3K/AKT signaling pathway regulates clathrin-mediated endocytosis and inhibition of this pathway suppresses the entry of different viruses such as reovirus [25, 26]. Additionally, it has been hypothesized that activation of the PI3K/AKT signaling pathway by SARS-CoV-2 contributes to induction of glucose uptake through glucose transporters (GLUTs), leading to increased glycolysis and viral replication in host cells [27]. Based on this evidence, the PI3K/AKT signaling pathway could be considered as a regulator of SARS-CoV-2 entry and replication in the host cells by regulating the clathrin-mediated endocytosis and glycolysis processes.

Among the downstream targets of the PI3K/AKT signaling pathway, mTOR and nuclear factor kappa B (NF-κB) are mostly linked to the pathogenicity of SARS-CoV-2. For instance, hyper-activity of NF-κB has been shown in SARS-CoV-2 infected cells [28]. Also, inhibition of mTOR followed by the PI3K/AKT signaling pathway suppression has been associated with inhibitory effects on the life cycle of SARS-CoV-2 in different studies [18, 19].

Anyway, in addition to probable direct activation of the PI3K/AKT pathway by SARS-CoV-2, there are several other routes that should be examined. Previously, we hypothesized the potential role of Ang II in PI3K/AKT signaling activation after SARS-CoV-2 induced infection [29]. In addition, elevated levels of pro-inflammatory cytokines may be involved in the PI3K/AKT signaling pathway in COVID-19 patients. For instance, elevated levels of interleukin (IL)-6, IL-1, TGF-β, and TNF-α have been detected in COVID-19 patients [26, 30]. On the other hand, the association between mentioned cytokines and the PI3K/AKT pathway has been examined in different studies [31–33].

Collectively, these data suggest that the PI3K/AKT signaling pathway can be over-activated in COVID-19 patients via direct or indirect mechanisms. The expression of mentioned factors’ receptors on different cells involved in coagulopathies will be discussed in the next sections.

### PI3K/AKT signaling pathway and platelet activation

Different PI3K isoforms, including all class I isoforms and PI3KC2a and PI3KC2b of class II isoform, are expressed in platelets, indicating the major role of this factor in platelet activity regulation [34]. In this regard, specific PI3K inhibition by PIK-75 and PI-103 has been shown to attenuate AKT activation, which eventually leads to suppression of platelet activation [35, 36]. In addition, it has been reported that pharmacologic inhibition of PI3Kα contributes to suppression of insulin-like growth factor-1 (IGF-1) induced AKT activation, inhibition of platelet activation and thrombus formation in cultured human platelets [37]. In a closer inspection, another study showed that PI3Kα inhibition augments thrombopoietin-mediated platelet activation and thrombus formation through restricting thromboxane A2 synthesis and extracellular signal-regulated kinase (ERK) phosphorylation [38]. Additionally, loureirin, a flavonoid extracted from dragon’s blood (a deep red resin secreted from *Dracaena*), has been shown to suppress platelet activation through PI3K/AKT signaling pathway inhibition [39].

AKT is the major downstream effector of PI3K in platelets. The PIP3-binding domain of AKT recruits it to the platelet plasma membrane, where it is activated by mammalian target of rapamycin (mTOR) and pyruvate dehydrogenase kinase 1 (PDK1) [40, 41]. AKT plays a central role in platelet activation as downstream target of a variety of receptors such as IGF-1 receptor [35]. All three AKT isoforms—AKT1, AKT2, and AKT3—are expressed in human and mouse platelets, and their role in platelet activation has been elucidated in different studies. Increased bleeding time, and impaired platelet aggregation, secretion and spreading in response to different agonists have been shown in AKT1 KO mice [42]. AKT2 is required for integrin activation and aggregation, and granule secretion induced by Gq induction by low doses of TxA2 and thrombin. Additionally, AKT2 KO platelets show diminished arterial thrombus generation [43]. Regarding AKT3, it has been proposed that it plays a crucial role in mediating granule secretion and platelet aggregation stimulated by TxA2 and thrombin [41]. Also, AKT3 is responsible for glycogen synthase kinase-3 (GSK-3) inactivation caused by thrombin, leading to arterial thrombosis in vivo [44, 45].

### PI3K/AKT pathway and coagulation factors’ expression and function

As described above, alterations in levels of several coagulation factors have been detected in samples from COVID-19 patients. Here we examine the role of PI3K/AKT signaling pathways in regulation of these factors’ expression and activity.

TF, a member of the cytokine receptor superfamily, is considered as an initiator of coagulation in different conditions such as blood vessel damage [46]. After mechanical or chemical damage of the vascular wall, TF is released to the blood and attaches to plasma factor VIIa to induce blood coagulation [47]. TF is expressed by a wide range of cells, the most important of which are endothelial cells, monocytes, neutrophils, and platelets [47]. Regarding the association between the PI3K/AKT signaling pathway and TF expression, there are several studies that should be examined. It has been shown that inhibition of the PI3K/AKT signaling pathway by wortmannin reduces both mRNA and protein levels of TF in breast cancer MDA-MB-231 cells [48]. Simvastatin, a HMG CoA reductase inhibitor, has been reported to suppress thrombin-induced TF expression via AKT inhibition in human endothelial cells [49]. In addition, PI3K/AKT signaling inhibition by PTEN has been shown to suppress TF expression in glioblastoma cells [50].

The other main coagulation factor is fibrinogen, which is cleaved by thrombin to generate fibrin clots [51]. It has been observed that dephosphorylation and inactivation of PI3K by ginsenoside Ro attenuates fibrinogen binding to glycoprotein IIb/IIIa, reflecting the intensification of thrombus in human platelets [52]. Also, it has been demonstrated that PI3K/AKT signaling inhibition by 8-ethoxy-2-(4-fluorophenyl)-3-nitro-2H-chromene leads to inhibition of fibrinogen binding of single platelet and thrombus formation in cultured human platelets [53].

Regarding thrombin, there are no data indicating the regulation of its expression by the PI3K/AKT signaling pathway. Nevertheless, numerous studies show that thrombin induces PI3K/AKT signaling activation, leading to activation of platelets and increased coagulation factor expression [52]. For instance, it has been elucidated that thrombin induces the PI3K/AKT signaling pathway, leading to increased expression of IL-6 and TF in MC3T3-E1 cells [54]. Also, it has been indicated that thrombin induces platelet activation through PI3K/AKT signaling activation [55]. These data suggest that elevated thrombin levels in COVID-19 patients may be another mechanism to induce blood coagulation by PI3K/AKT pathway activation.

### The association between Ang II, the PI3K/AKT pathway and coagulopathies

Ang II is a vasoconstrictor factor which plays a crucial role in vascular disease. In addition to its physiologic activity, Ang II is known for its pro-inflammatory and pro-coagulatory properties. Elevated levels of Ang II have been detected in patients with COVID-19 [56, 57]. This can be explained by the mechanism of SARS-CoV-2 entry into host cells. One of the main receptors of SARS-CoV-2 is angiotensin converting enzyme 2 (ACE2), which converts Ang II to angiotensin 1–7 in physiological conditions. Once SARS-CoV-2 has attached to ACE2, its endocytosis to the host cell occurs and the amount of ACE2 on the cell surface decreases. This process leads to increased Ang II levels due to reduced Ang II conversion to angiotensin 1–7 [29]. To describe the role of Ang II in expression of coagulation factors and platelet activity, it should first be noted that the expression of its receptor, Ang II receptor type I (AT1R), has been detected in platelets, endothelial cells, and monocytes [58–60]. Regarding the role of Ang II in blood coagulation, it has been shown that Ang II administration to mice enhances thrombosis in arterioles and/or venules [58]. Additionally, it has been indicated that Ang II infusion in mice models contributes to platelet activation, leading to cardiac fibrosis [61]. Also, low concentrations of Ang II have been shown to enhance adrenaline-induced platelet aggregation [62].

In addition to the role of Ang II in platelet activation, its role in TF expression has been examined in different studies. In this case, AT1R blockers have been shown to reduce TF expression in hypertensive patients [63]. Also, angiotensin-converting enzyme (ACE) inhibitors, which reduce Ang II production, have been demonstrated that suppress TF expression and activity in cultured monocytes [60].

As described, the PI3K/AKT signaling pathway is one of the main downstream effectors of AT1R, and the Ang II-AT1R axis contributes to activation of this pathway [64]. To prove this claim that PI3K/AKT signaling mediates Ang II-induced coagulation factor expression, it can be referred to a study that shows that Ang II induces TF expression through activation of NF-ҡB, one of the main downstream targets of the PI3K/AKT pathway [65]. Also, it has been elucidated that simvastatin, a PI3K/AKT signaling inhibitor, suppresses Ang II-induced TF expression in cultured rat aortic endothelial cells [66].

Collectively, these data suggest that elevated levels of Ang II in COVID-19 patients may contribute to the PI3K/AKT signaling over-activation, leading to induction of platelet activation and coagulation factor expression.

### The association between inflammatory cytokines, the PI3K/AKT pathway and coagulopathies

The other PI3K/AKT signaling pathway stimuli which have been increased in COVID-19 patients are inflammatory cytokines, including IL-6, IL-1, TGF-β, and TNF-α. These factors are closely associated with blood coagulation by different mechanisms. IL-6 and IL-1β have been shown to increase hypercoagulability of whole blood by a direct effect on platelets [67]. Also, increased TF expression by IL-6, IL-1β, and TNF-α has been elucidated in porcine aortic endothelial cells [68]. Since the IL-6 receptor is present in endothelial cells and its activation by IL-6 contributes to the PI3K/AKT pathway activation [69], it can be concluded that the effect of IL-6 on TF expression is mediated by this pathway. Regarding IL-1, on one hand, its receptor is expressed in platelets [70] and, on the other hand, it has been shown that the PI3K/AKT signaling pathway mediates its effects in different cells [71]. In this case, it has been demonstrated that IL-1β binding to IL-1 receptor in platelets induces their activation through NF-ҡB, one of the main downstream targets of AKT [72]. In addition to IL-1, up-regulation of TF expression in endothelial cells by TNF-α through NF-ҡB activation suggests that PI3K/AKT signaling mediates its effect on TF expression [73]. TGF-β, the other increased inflammatory cytokine in COVID-19 patients, has been linked to blood coagulation. It has been indicated that TGF-β induces TF expression in human lung fibroblasts through PI3K/AKT signaling activation [74].

**Fig. 1:**
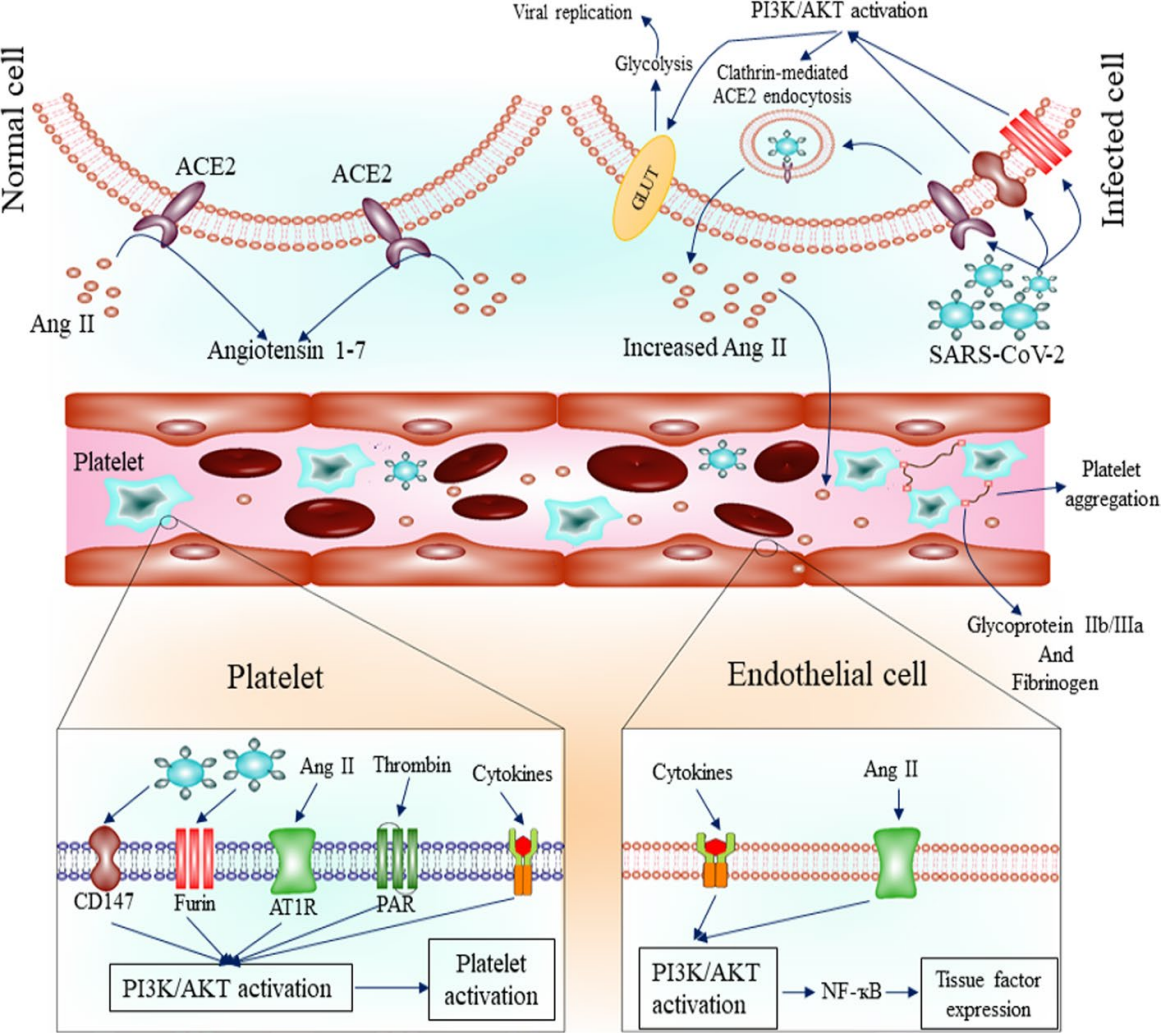
The role of the PI3K/AKT signaling pathway in blood coagulation in SARS-CoV-2 infection. Endocytosis of ACE2 followed by SARS-CoV-2 attachment contributes to increased Ang II levels. In addition, elevated levels of thrombin and inflammatory cytokines, such as TNF-α, IL-6, IL-1, and TGF-β, have been detected during SARS-CoV-2 infection. Mentioned factors can activate the PI3K/AKT signaling pathway in platelets, leading to its over-activation and clot formation. In addition, PI3K/AKT signaling activation by mentioned factors contributes to increased tissue factor expression in endothelial cells. ACE2: angiotensin converting enzyme 2; Ang II: angiotensin II; IL: interleukin; PI3K: phosphatidyl-inositol-3-kinases; TGF-β: transforming growth factor β; TNF-α: tumor necrosis factor α

Overall, these data suggest the role of elevated inflammatory cytokines in COVID-19 patients in blood coagulation which may be mediated by the PI3K/AKT signaling pathway. Figure 1 presents the role of the PI3K/AKT signaling pathway in blood coagulation during SARS-CoV-2 infection.

## Conclusion

Based on the existence evidence, it can first be noted that the PI3K/AKT signaling pathway may be over-activated during SARS-CoV-2 infection by two mechanisms: (1) direct activation through its receptors, furin and CD147, on different cells such as platelets and (2) indirect activation via elevated levels of Ang II, inflammatory cytokines, and thrombin. On the other hand, due to the central role of the PI3K/AKT pathway in platelet activation and expression of coagulation factors, it can be said that this pathway may be a therapeutic target to reduce coagulation complications in COVID-19 patients.

## Data Availability

Not applicable.

## Abbreviations

ACE2: Angiotensin converting enzyme 2
Ang II: Angiotensin II
IL: Interleukin
PI3K: Phosphatidyl-inositol-3-kinases
TGF-β: Transforming growth factor β
TNF-α: Tumor necrosis factor α
